# Association between 1400 metabolites and IgA nephropathy: A Mendelian randomization analysis

**DOI:** 10.1097/MD.0000000000043353

**Published:** 2025-07-25

**Authors:** Chenxin Wang, Yanran Li, Linyu Zhong, Na Sun, Denggui Luo, Yuanzhao Xu, Airong Qi

**Affiliations:** a The Fourth Clinical Medical College of Guangzhou University of Chinese Medicine, Shenzhen, Guangdong, China.

**Keywords:** causal effect, FinnGen, IgA nephropathy, Mendelian randomization, serum metabolites

## Abstract

IgA nephropathy (IgAN) is the leading cause of end-stage renal disease, although its mechanisms remain incompletely understood. Previous studies have identified metabolites associated with IgAN, but their causal relationships require further investigation. This study employed a 2-sample Mendelian randomization (MR) approach to assess the causal relationships between 1400 serum metabolites and IgAN. Causal effects between these metabolites and IgAN were estimated using the inverse-variance weighted method. Additional analyses, including MR-Egger regression, weighted median, simple mode, and weighted mode methods, were conducted to refine and validate these findings. Pleiotropy and heterogeneity tests were also performed. The initial analysis identified 9 known and 4 novel metabolites associated with IgAN. Notably, Acisoga was found to increase the risk of IgAN, whereas serine exhibited a protective effect; both findings were confirmed by robust statistical tests (*P* < .05). This initial MR analysis highlights 2 metabolites significantly linked to IgAN, providing valuable insights into the disease’ s underlying mechanisms for clinical research. Further investigation is needed to validate these findings.

## 1. Introduction

Currently, IgA nephropathy (IgAN), with a global incidence rate of 1 to 2.5 cases per 100,000 individuals, is recognized as the leading form of chronic glomerulonephritis. Alarmingly, 40% of those affected by IgAN are projected to progress to end-stage renal disease within 2 decades,^[1–3]^ posing a significant threat to public health and underscoring IgAN as a critical challenge that urgently requires effective solutions. However, as a kidney disease primarily characterized by the deposition of immune complexes, hematuria is a key indicator of IgAN, particularly common in young people. In adults, however, macroscopic hematuria or proteinuria is more likely to occur and is often detected incidentally during health checkups.^[4–6]^ This can result in IgAN being underdiagnosed, thereby increasing the risks of kidney failure and mortality.

Nonetheless, the underlying mechanisms of IgAN are not fully understood, with the prevailing explanation being the “multi-hit” hypothesis. In patients with IgAN, there is a notable increase in galactose-deficient immunoglobulin A1 (Gd-IgA1) levels, which is recognized as an autoantigen by the immune system. This recognition leads to the formation of specific antibodies that bind to Gd-IgA1, resulting in immune complexes (anti-Gd-IgA1 IgG or IgA antibodies).^[7,8]^ These complexes accumulate in the mesangial regions of the kidneys, triggering inflammation and glomerular damage. The production pathways of Gd-IgA1, the initiation of autoreactive antibodies by it, the mechanisms by which these immune complexes deposit in the mesangial areas, and the subsequent cascade of biological responses remain under investigation.^[9]^

Metabolomics, the comprehensive analysis of biological substances within an organism, holds significant promise for uncovering disease mechanisms, identifying therapeutic targets, and discovering biomarkers. This approach is increasingly being applied in IgAN research, offering insights into distinctive metabolic alterations in patients’ blood and urine samples. Notably, changes in carbohydrates, lipids, and amino acid metabolism, including compounds such as 8-hydroxyguanosine, phenylalanine, and glycine, have been identified.^[10]^ These metabolomic profiles could play a crucial role in enhancing diagnosis, improving patient outcomes, and developing novel treatment strategies.^[9,11,12]^ However, the inherent complexity of observational studies, variability in testing methodologies, and discrepancies across different studies pose significant challenges in elucidating the interactions between these metabolites and IgAN. Given the scarcity of prospective studies, there is an urgent need for more in-depth investigations and validation. Therefore, a detailed analysis of the causal relationships between IgAN and its associated metabolites is essential to enhance our understanding and inform future therapeutic approaches.

Mendelian randomization (MR) uses genetic variations as instrumental variables (IVs) to estimate causal links between exposure factors and disease outcomes when randomized controlled trials are not feasible. This method emulates randomized controlled trials by leveraging the natural randomness in genetic distribution to simulate random grouping within a population, effectively minimizing confounding factors.^[13,14]^ This innovative approach offers a unique pathway for exploring the causal connections between serum metabolites and IgAN.

This research aims to investigate the potential causal links between blood metabolites and IgAN, providing fresh perspectives that could inform new preventive and therapeutic strategies for the disease.

## 2. Materials and methods

### 2.1. Study design

**Figure 1.**
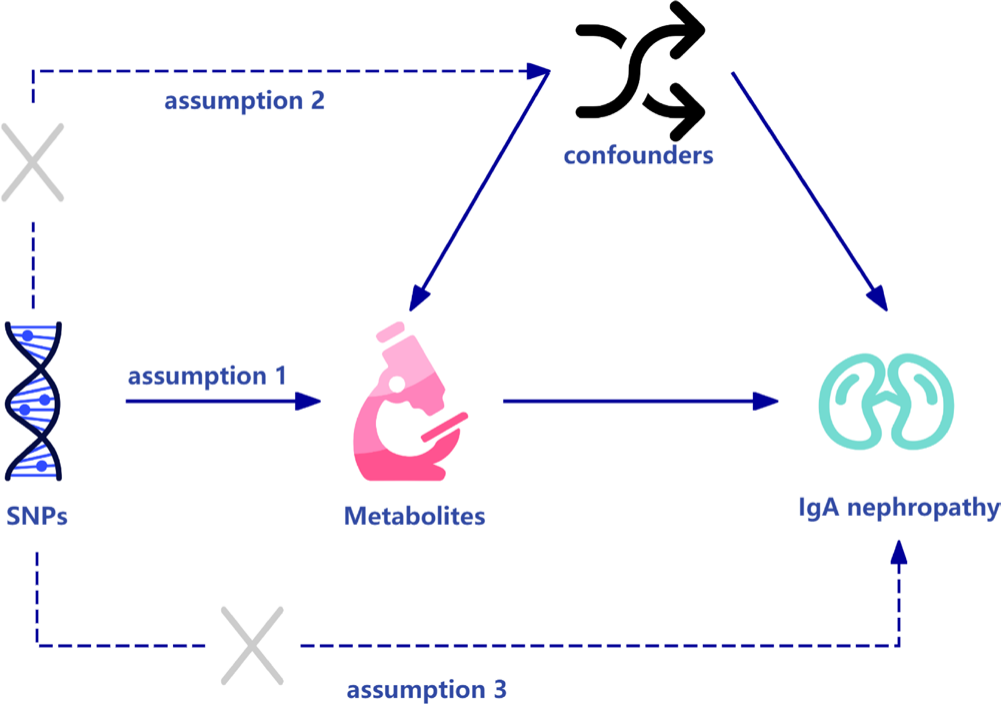
The principle of MR study. Assumption 1: Genetic variation exhibits a close association with the exposure (metabolites). Assumption 2: Genetic variation is independent of confounding factors, ensuring unbiased results. Assumption 3: Genetic variation influences the outcome (IgAN) exclusively through the exposure pathway. IgAN = IgA nephropathy, MR = Mendelian randomization.

This study will analyze data from a European population Genome-Wide Association Study (GWAS) to determine the causal effects of 1400 metabolites on IgAN using the 2-sample MR approach. This method relies on 3 critical assumptions: First, there must be a strong correlation between the exposure factors (metabolites) and the single-nucleotide polymorphisms (SNPs) serving as IVs. Second, SNPs must be independent of other confounders due to the random allocation of genes, which is unaffected by external variables. Third, the influence of the SNPs on the outcomes must be mediated solely through the exposure factors, without any alternative pathways. With these assumptions in place, and the relationships among the IVs, exposure factors, and outcomes established, we can infer the causal relationships between the exposure factors and the outcomes^[15]^ (Fig. 1).

### 2.2. Data sources

The GWAS dataset for human metabolites used in this research was provided by Chen et al in a 2023 publication.^[16]^ This dataset offers an extensive analysis of GWAS conducted on 1091 blood metabolites and 309 ratios of these metabolites, sourced from the GWAS Catalog database (ebi.ac.uk) under the catalog numbers GCST90199621-GCST90201020. This study utilized data from 8299 unrelated individuals of European ancestry from the Canadian Longitudinal Study of Aging cohort. Genotyping was performed using the Affymetrix Axiom platform, followed by imputation with the TOPMed reference panel. Standard pre-GWAS quality control was applied, including removal of low-quality SNPs (MAF ≤ 0.1%, imputation quality ≤ 0.3, or missing rate ≥ 0.1). To control for population stratification in the association analyses, Chen et al adjusted for age, sex, hours since last meal/drink, genotyping batch, and the first 10 genetic principal components (PC). For IgAN, the GWAS data were obtained from the FinnGen database (https://www.finngen.fi/en/access_results), identified by data ID finngen_R10_N14_IGA_NEPHROPATHY. This dataset includes records for 653 patients with IgAN and 411,528 healthy individuals, verified through discharge or mortality ICD codes [N08.2*D89.80]. The FinnGen project is a substantial genetic study aimed at investigating the connections between genetic information and health outcomes in the Finnish population.^[17]^ To control for population stratification, the FinnGen consortium implemented a rigorous multi-stage PC Analysis pipeline. This involved iterative linkage disequilibrium pruning of variants, merging with 1000 Genomes Project data for initial outlier detection using a Bayesian algorithm, followed by a FinnGen-specific PC Analysis and projection of 1000 Genomes European and Finnish samples. Mahalanobis distance was used to refine Finnish ancestry and exclude further outliers, resulting in a set of unrelated individuals of Finnish ancestry for whom PCs were calculated and used as covariates in the GWAS analyses. This process aimed to robustly account for the unique genetic substructure of the Finnish population and minimize confounding. All original studies received ethical review approval and obtained informed consent. Details regarding the sample sizes, ancestry, and SNP counts of the 2 summary-level GWAS datasets are summarized in Table 1. The metabolite GWAS and the IgAN GWAS are distinct consortia, reducing the likelihood of substantial direct participant overlap. This study primarily conducted its analysis using the TwoSampleMR package in R software (version 4.3.2, R Foundation for Statistical Computing, Vienna, Austria), with additional data visualization and processing performed using the ggplot2 package.

**Table 1:** Characteristics of Genome-Wide Association Study (GWAS) summary statistics used in the Mendelian randomization analysis.

Characteristic	Exposure: serum metabolites	Outcome: IgAN
Source	Chen et al. (2023) Nat Genet. 55:44-53^[16]^	FinnGen Consortium (Release R10)^[17]^
Database ID(s)	GCST90199621-GCST90201020	finngen_R10_N14_IGA_NEPHROPATHY
Number of traits analyzed in source	1091 blood metabolites + 309 metabolite ratios	IgA nephropathy
Sample size (for source GWAS)	8299 unrelated individuals	412,181 (653 cases, 411,528 controls)
Participant ancestry (in source GWAS)	European ancestry (from Canadian Longitudinal Study on Aging - CLSA)	European ancestry (Finnish population)
No. of SNPs in original GWAS	Approx. 15.4 million SNPs (post-imputation & QC)	Approx. 21.3 million SNPs

GWAS = Genome-Wide Association Study, IgAN = IgA nephropathy, SNP = single-nucleotide polymorphism.

### 2.3. IV selection

In selecting SNPs associated with 1400 metabolites, we applied stringent criteria, specifically a *P* value of <5 × 10^−8^.^[18]^ To maintain independence among the SNPs, in accordance with Mendel second law, we established linkage disequilibrium thresholds of *r*^2^ < 0.001 and a physical distance >10,000 kilobases.^[19]^ SNPs that met these criteria were used as IVs in further analysis. The *F* value served as a measure of the correlation between an IV and the exposure of interest, defined by the formula *F* = (*β*/SE)^2^. Based on our literature review, variables with an *F* value below 10 were considered weak IVs.^[20]^ To uphold the foundational premise of MR, we excluded these weak IVs from our study, all IVs ultimately included in our MR analyses for each metabolite robustly exceeded this F-statistic threshold of 10, indicating that strong genetic instruments were used, thereby minimizing the potential for weak instrument bias. A detailed list of the selected instrumental SNPs for each metabolite, along with their individual F-statistics, is provided in Tables S1 and S2, Supplemental Digital Content, https://links.lww.com/MD/P498.

### 2.4. MR analysis

We analyzed data using the TwoSampleMR package in R, focusing on the inverse-variance weighted (IVW) method to assess correlations, a standard approach that excludes the intercept and utilizes inverse outcome variance for fitting.^[21]^ Despite its utility, potential biases from pleiotropy and confounding effect sizes remain a concern. To address this, we incorporated MR-Egger regression and the weighted median method as additional checks for validating causal relationships. These methods were applied to explore links between metabolites and IgAN. Consistency in causal directions across these models suggests a stable causal relationship between the exposure and outcome. Notably, MR-Egger differs from IVW by considering the intercept in its regression, while the weighted median method adjusts for variations in estimation precision.^[22]^ The weighted median approach yields precise estimates, provided that at least half of the IVs are valid.^[23]^ We considered a *P* value under 0.05 as significant. Moreover, to account for multiple testing across metabolites showing such nominal significance, we applied the Benjamini–Hochberg false discovery rate (FDR) procedure, with an FDR-adjusted *P* value (*q* value) < 0.05 considered as evidence of a robust, statistically significant association. Additionally, to minimize Type II errors and to assess their frequency, we calculated statistical power using the mRnd online tool, setting the Type I error parameter at *α* = 0.05.^[24]^

### 2.5. Sensitivity analyses

We assessed horizontal pleiotropy using the MR-Egger intercept test and the MR-PRESSO method to identify and exclude pleiotropic outliers, thereby enhancing the robustness of our analysis.^[25,26]^ A *P* value >0.05 indicates the absence of significant horizontal pleiotropy. We also evaluated heterogeneity, which represents the variability in the causal effects of SNPs related to the exposure. Significant heterogeneity (*P* <  .05), as assessed by the Cochran *Q* test, suggests instability in these causal effects.^[25]^ To further ensure the robustness of our results, we employed the “leave-one-out” sensitivity analysis, in which each SNP was sequentially excluded, and the meta-effect of the remaining SNPs was recalculated.^[27]^ This method helps to ensure that our findings are not unduly influenced by any individual SNP. A significant MR Steiger test result (*P* < .05) confirms that the exposure precedes the outcome, thereby verifying the correct directional relationship.^[28]^

## 3. Results

**Figure 2.**
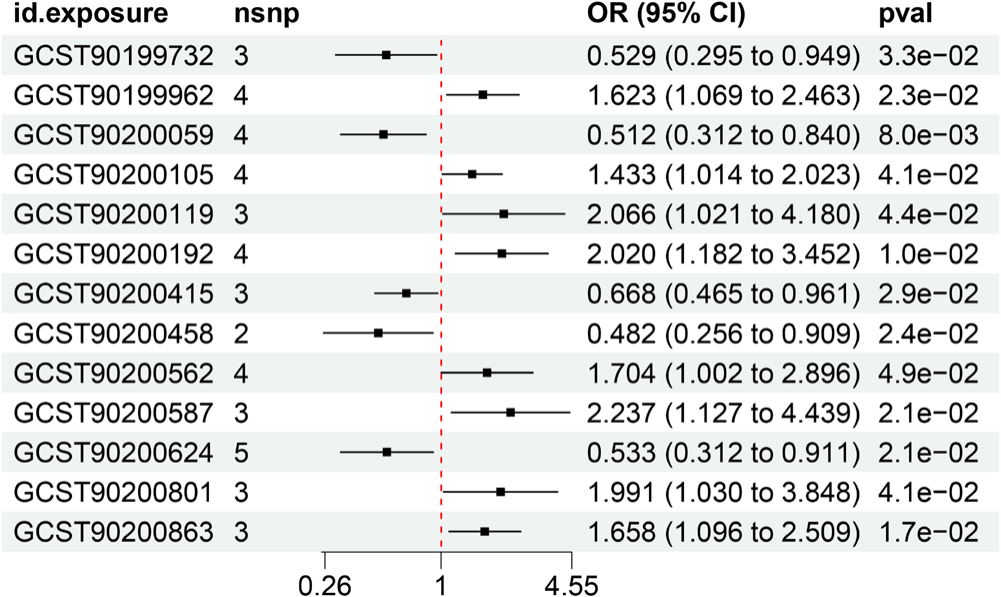
Forest plots demonstrated the causal associations between serum metabolites and IgAN. The analysis method is IVW. IgAN = IgA nephropathy, IVW = inverse-variance weighted.

**Figure 3.**
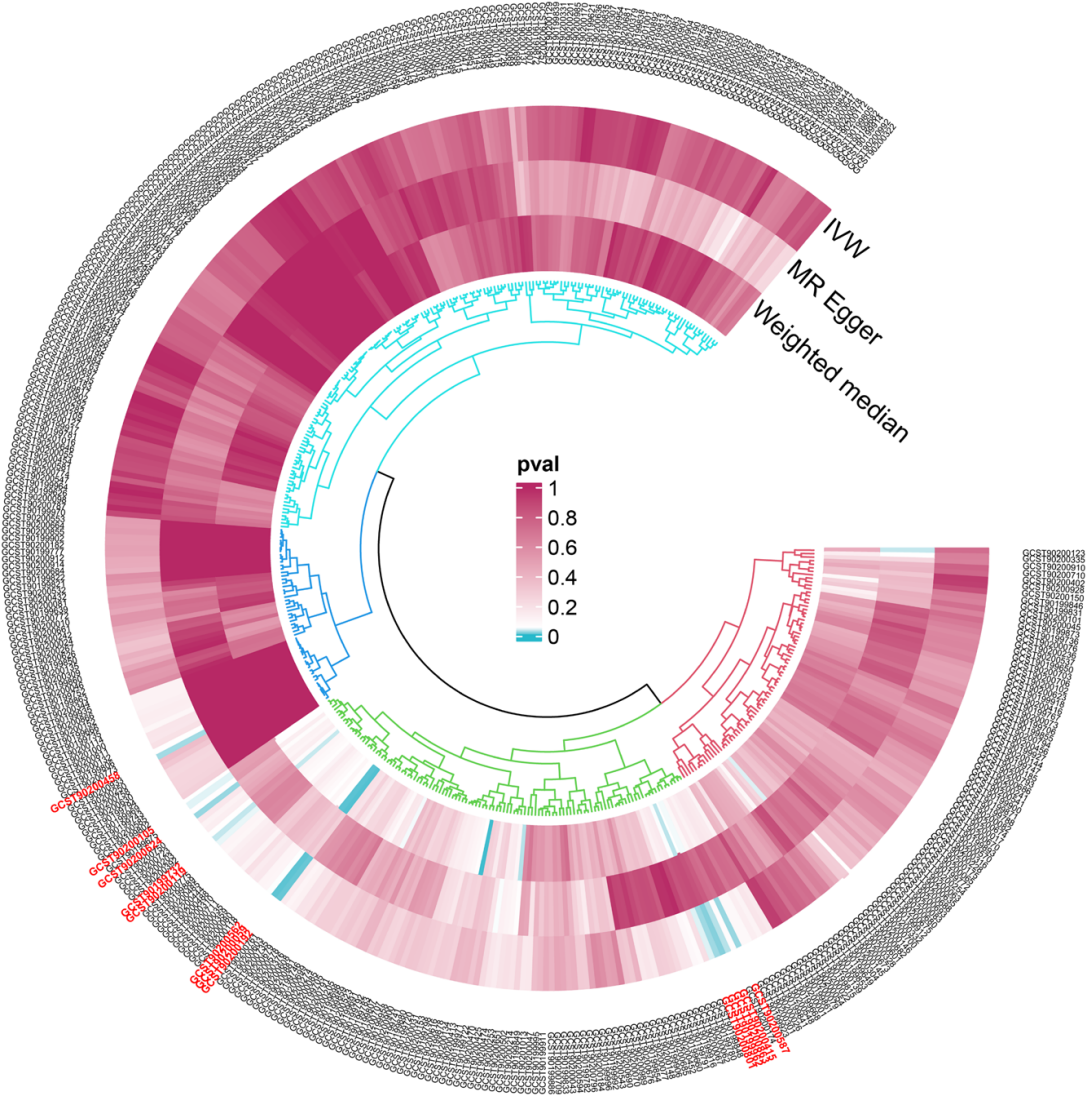
Heatmap showing the causal estimates of serum metabolites on IgAN aging in the primary analyses with IVW, MR-Egger, and weighted median methods. IgAN = IgA nephropathy, IVW = inverse-variance-weighted, MR = Mendelian randomization.

Using the IVW method, we initially identified 13 metabolites linked to IgAN, including 9 known and 4 unknown metabolites (Table S3, Supplemental Digital Content, https://links.lww.com/MD/P498). Among the known metabolites, increases in 6 are correlated with a higher risk of IgAN, including Acisoga, glycosyl-n-behenoylsphingadienine (d18:2/22:0), glycosyl ceramide (d18:1/23:1, d17:1/24:1), n,n-dimethylalanine, the ratio of 5-methylthioadenosine to phosphate, and the ratio of phosphate to L-serine. Conversely, the following metabolites are associated with a reduced risk of IgAN: 1-stearoyl-gpc (18:0), behenoyl dihydrosphingomyelin (d18:0/22:0), and L-serine (Figs. 2 and 3, Figures S1 and S2, Supplemental Digital Content, https://links.lww.com/MD/P497).

**Figure 4.**
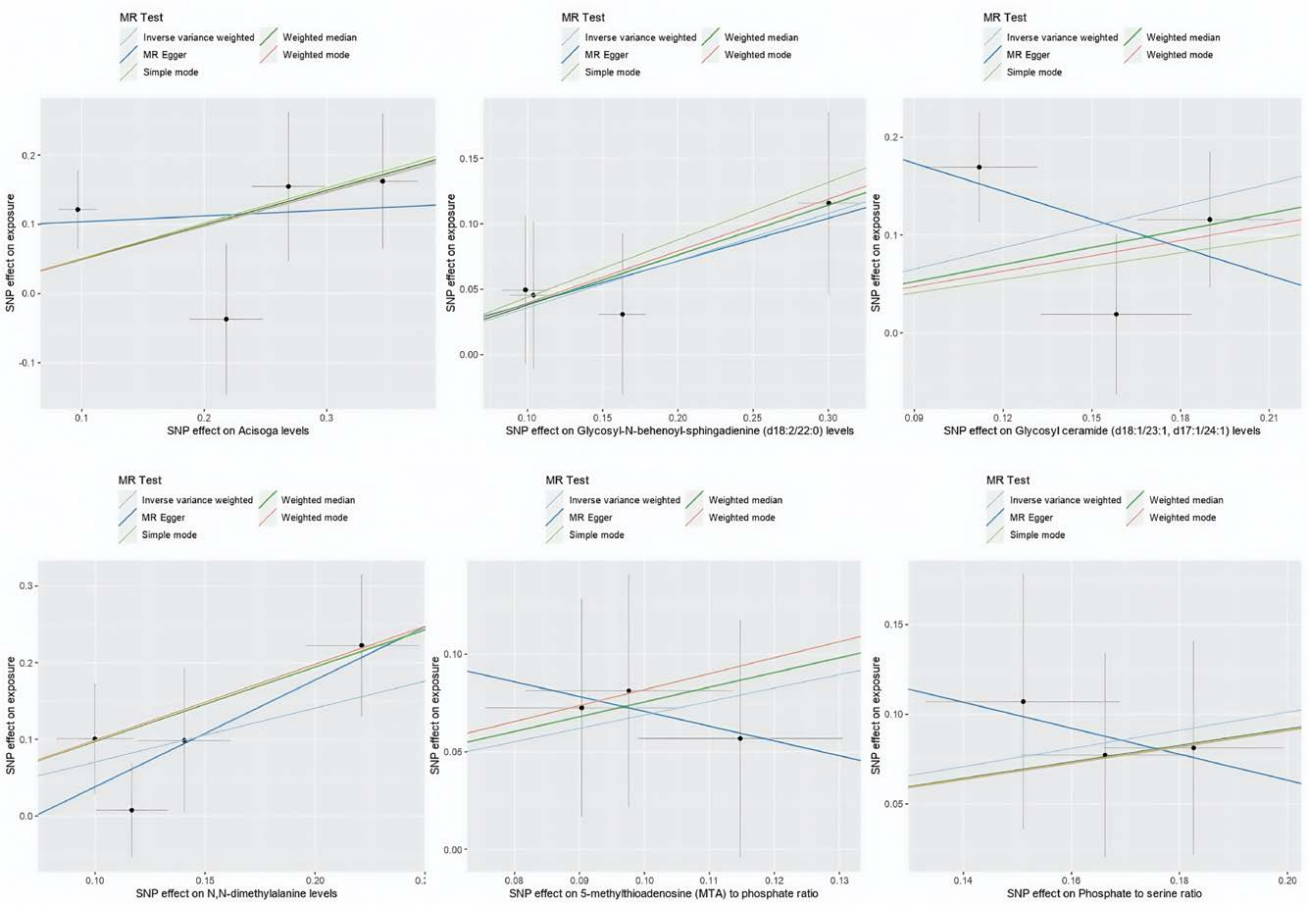
Scatterplot indicating the 6 metabolites as risk factors for IgAN in a MR analysis with statistical significance (*P* < .05). IgAN = IgA nephropathy, MR = Mendelian randomization.

**Figure 5.**
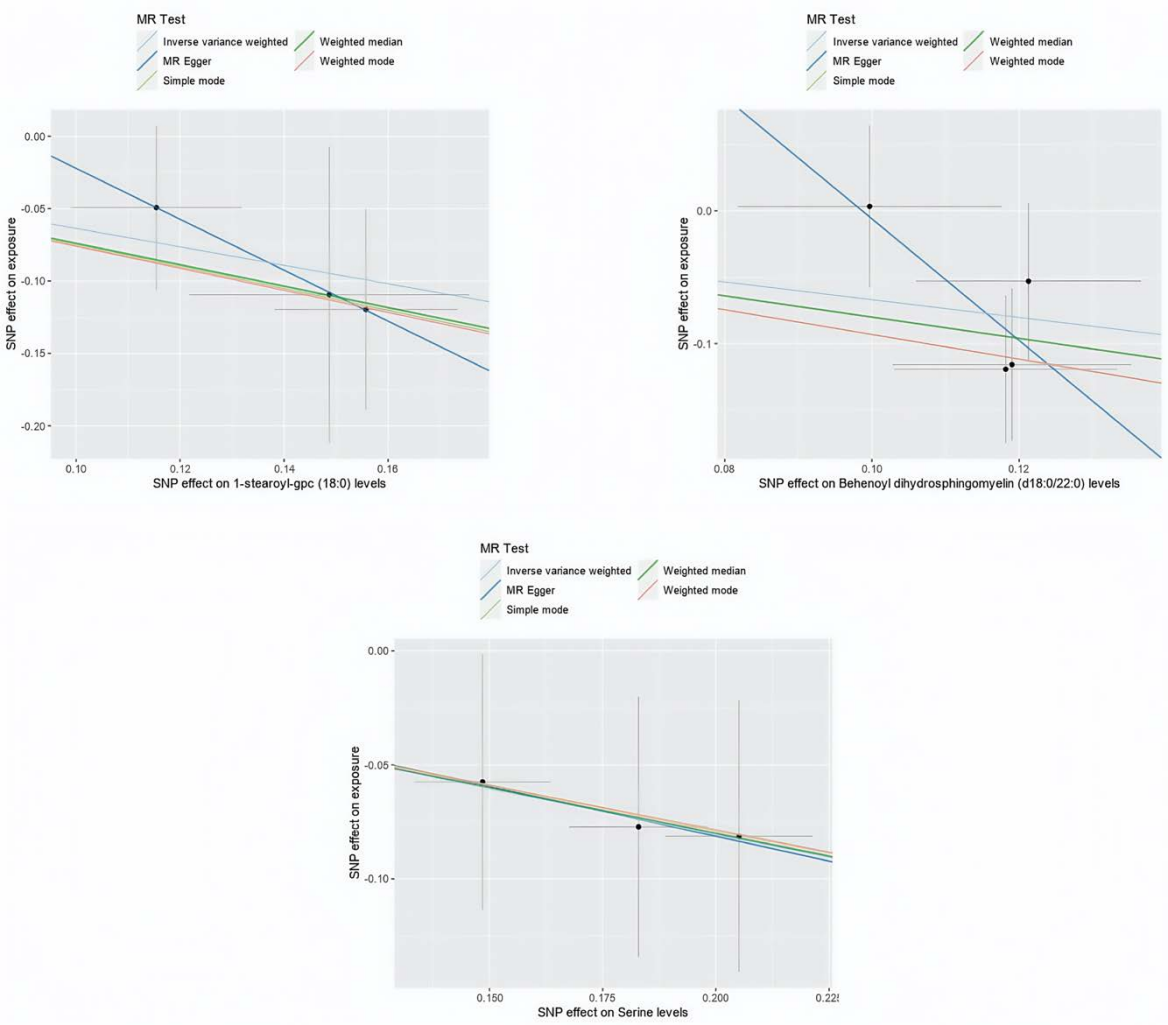
Scatterplot indicating the 3 metabolites as protective factors for IgAN in a MR analysis with statistical significance (*P*<0.05). IgAN = IgA nephropathy, MR = Mendelian randomization.

All *F*-statistics exceeded 10, indicating that no weak instruments were used. To enhance the reliability of our findings, we included only metabolites supported by at least 3 SNPs in this study. To rule out the effects of other confounding factors, we assessed the causal relationships between blood metabolites and IS using multiple approaches, including IVW analysis, MR-Egger regression, the weighted median method, simple mode, and weighted mode (Figs. 4 and 5).

**Figure 6.**
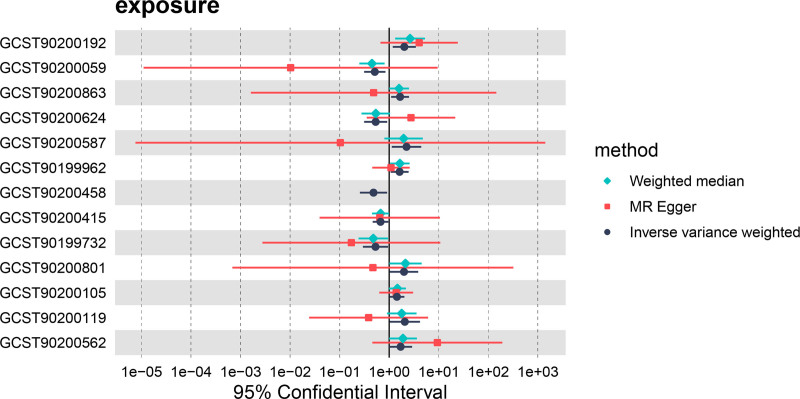
Forest plots showed the causal associations between serum metabolites and IgAN. The analysis method is IVW, weighted median and MR-Egger. IgAN = IgA nephropathy, IVW = inverse-variance weighted, MR = Mendelian randomization.

We also performed tests for pleiotropy and heterogeneity (Tables S4 and S5, Supplemental Digital Content, https://links.lww.com/MD/P498). The findings indicated that among the 9 identified metabolites, 5 showed significant associations (*P* <  .05) in at least 2 MR models (Table 2). For the unknown metabolites, X11315 (ID:GCST90200458) lacked sufficient SNPs, limiting causality assessments to the IVW method only (Fig. 6).

**Table 2:** *P* values for 5 MR models (IVW, MR-Egger, weighted median, simple mode, weighted mode).

Metabolites	NSNP	IVW *P* value	Egger *P* value	Weighted median *P* value	Simple mode *P* value	Weighted mode *P* value
1-Stearoyl-gpc (18:0)	3	.033	.557	.031	.218	.210
Acisoga	4	.023	.868	.037	.237	.153
Glycosyl-N-behenoyl-sphingadienine (d18:2/22:0)	4	.041	.502	.055	.203	.184
Glycosyl ceramide (d18:1/23:1, d17:1/24:1)	3	.044	.621	.073	.397	.298
N,N-dimethylalanine	4	.010	.268	.004	.155	.112
Serine	3	.029	.811	.047	.251	.250
5-Methylthioadenosine	3	.041	.858	.064	.225	.246
Phosphate to serine ratio	3	.017	.845	.061	.268	.239
Behenoyl dihydrosphingomyelin (d18:0/22:0)	4	.008	.319	.006	.116	.112

IVW = inverse-variance weighted, NSNP = non single-nucleotide polymorphism.

Horizontal pleiotropy analysis conducted using MR-Egger intercepts indicates that all 9 known metabolites have *P* values above 0.05, suggesting a minimal risk of horizontal pleiotropy. Additionally, tests for heterogeneity using Cochran *Q* indicated no significant heterogeneity among these metabolites, as all had IVW and MR-Egger *Q*-derived *P* values exceeding 0.05. Some metabolites, with fewer than 4 SNPs, were only partially assessed for horizontal pleiotropy using the MR-PRESSO method, which found no outliers (*P* >  .05). Detailed data are provided in the Supplementary Materials. However, after applying multiple FDR corrections, as detailed in the Methods section (using the Benjamini–Hochberg procedure with an FDR-adjusted *q* value < 0.05 threshold), only 3 metabolites remained significantly associated with IgAN (Table S8, Supplemental Digital Content, https://links.lww.com/MD/P498). These 3 consistently demonstrated the same causal relationship direction across all 4 analytical methods.

**Figure 7.**
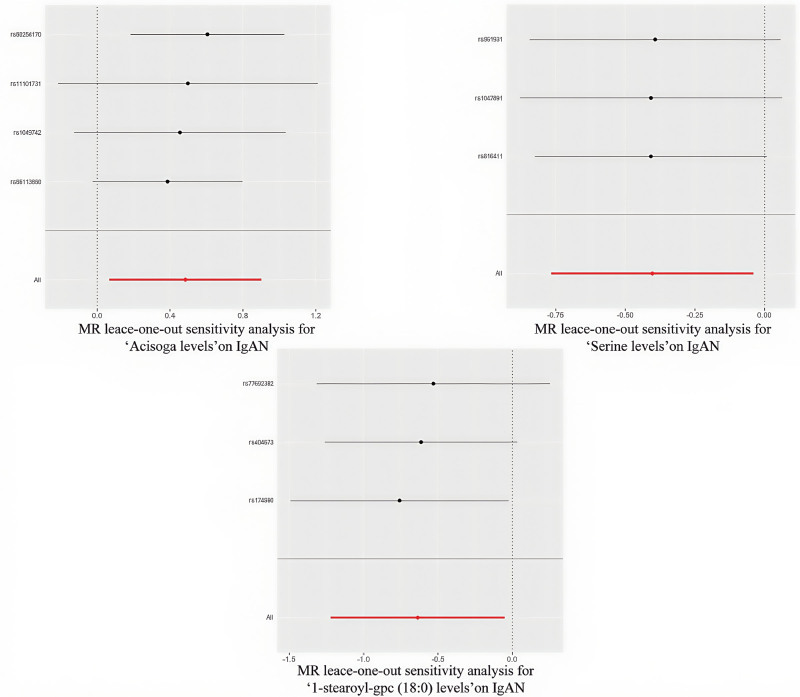
MR leave-one-out sensitivity analysis for the 3 metabolites on IgAN. IgAN = IgA nephropathy, MR = Mendelian randomization.

A leave-one-out sensitivity analysis showed that no individual SNP had a dominant influence on the overall results (as illustrated in Fig. 7, Figures S3 and S4, Supplemental Digital Content, https://links.lww.com/MD/P497). Directionality tests using MR Steiger found that the levels of 1-stearoyl-gpc (18:0) did not significantly influence the causal direction and were therefore excluded (Table S7, Supplemental Digital Content, https://links.lww.com/MD/P498). This exclusion helped confirm the potential causal direction of the other 2 metabolites, with significant findings (*P* <  .05) detailed in Table 3 and Figure S5, Supplemental Digital Content, https://links.lww.com/MD/P497.

**Table 3:** The results of heterogeneity test and pleiotropy test and Steiger test for candidate blood metabolites and IgAN.

	Heterogeneity test			
Metabolites	MR-Egger Q_pval	IVW Q_pval	Pleiotropy test_pval	Steiger test_pval
1-Stearoyl-gpc (18:0)	0.986	0.865	0.686	0.307
Acisoga	0.322	0.325	0.412	0.019
Glycosyl-N-behenoyl-sphingadienine (d18:2/22:0)	0.864	0.960	0.935	0.007
Glycosyl ceramide (d18:1/23:1, d17:1/24:1)	0.212	0.143	0.436	0.308
N,N-dimethylalanine	0.464	0.540	0.512	0.252
Serine	0.941	0.997	0.985	0.015
5-Methylthioadenosine	0.855	0.894	0.738	0.367
Phosphate to serine ratio	0.813	0.889	0.745	0.061
Behenoyl dihydrosphingomyelin (d18:0/22:0)	0.550	0.482	0.377	0.262

IgAN = IgA nephropathy, IVW = inverse-variance weighted, MR = Mendelian randomization.

Ultimately, after thorough screening, we identified 2 metabolites that appear to have causal links with IgAN. Acisoga is significantly positively associated with IgAN, indicating that higher levels may increase the risk of the disease. Conversely, serine shows a negative causal relationship, suggesting that higher levels might reduce the risk of IgAN.

## 4. Discussion

In this study, we utilized the latest GWAS data on metabolites and applied the 2-sample MR method to explore the causal relationships between blood metabolites and IgAN. By carefully selecting SNPs and conducting sensitivity analyses, we aimed to reduce confounding influences and strengthen the evidence for a causal connection. We also referenced previous research by Wang et al,^[29]^ which included a study on metabolites and IgAN but involved fewer metabolite types and did not yield positive findings regarding their relationship with IgAN. Consequently, our study expanded on this area by incorporating GWAS research on 1400 metabolites, thereby broadening the scope of investigation in this field.

Acisoga, identified as n-(3-acetamidopropyl)pyrrolidin-2-one (CAS number 106692-36-8), has emerged in metabolomic analyses as a potential factor associated with obesity, atrial fibrillation, and ventricular dysfunction. However, its link to IgAN remains unexplored.^[30,31]^ Acisoga is derived from the metabolic breakdown of spermine and putrescine, which are integral to polyamine metabolism. Specifically, arginine metabolism produces putrescine, which is converted to spermine by spermine synthase. Spermine and putrescine are then transformed into N1-acetylspermine by spermidine/spermine N1-acetyltransferase (SSAT) or polyamine oxidase, and subsequently into Acisoga through amine oxidase activity.^[32]^ Polyamines are essential for cell growth, differentiation, and responses to environmental stress. Elevated levels of SSAT, an enzyme critical for polyamine breakdown, have been observed in patients with kidney diseases or failure.^[33]^ Animal models have shown that SSAT levels peak in the kidneys after ischemic reperfusion injury.^[34]^ Additionally, in cisplatin-induced kidney damage models, increased expression of polyamine degradation enzymes has been observed, with significant mitigation of renal damage and fibrosis noted in enzyme-deficient models.^[35]^ Furthermore, the polyamine pathway is notably implicated in immunological responses, particularly influencing Th17 cells, as evidenced by various studies, including scRNA-seq and clinical research.^[36]^ These pathways are known to regulate crucial immune modulators like Foxp3, which is associated with renal fibrosis and adverse renal outcomes.^[36,37]^ In IgAN, an imbalance between Th17 cells and regulatory T cells is prevalent,^[38]^ Moreover, studies have shown that IgA immune complexes can selectively activate CD103 + dendritic cells by interacting with the IgA Fc receptor, enhancing Th17 cell responses and thereby promoting the production of inflammatory cytokines TNF, IL-1β, and IL-23.^[39]^ Thus, as a final product of polyamine metabolism, Acisoga may primarily contribute to the onset and progression of IgAN through its toxic effects on tubular epithelial cells, leading to tubular atrophy and interstitial fibrosis, and by modulating Th17 cell functions to induce inflammatory responses in IgAN. Consequently, measuring serum concentrations of Acisoga may assist in the early identification and assessment of the histological state in IgAN patients. Moreover, as a critical component of polyamine metabolism, inhibiting the relevant degrading enzymes might mitigate kidney damage and slow disease progression, though the precise mechanisms involved require further investigation.

L-serine, also known as Ser or S, has the chemical formula C_3_H_7_NO_3_, a molecular weight of 105.09 g/mol, and is listed under CAS number 56-45-1. Although not essential, this amino acid is produced in small amounts in the brain and primarily in the liver and kidneys. It is synthesized from 1-carbon units and glycine and then released into the blood. L-serine is crucial for carbohydrate metabolism, lipid synthesis, and protein synthesis, playing a central role in these metabolic pathways. It readily transforms into 1-carbon units and acts as a catalyst in the synthesis of key molecules such as purines, heme, thymidine, and creatine, which influence inflammation, immune responses, redox balance, and mitochondrial function.^[40]^ According to the literature, abnormalities in serum L-serine levels are commonly observed in neurological conditions such as diabetic peripheral neuropathy and Alzheimer disease. This is attributed to the fact that the liver and kidneys are essential organs for maintaining L-serine balance. Therefore, disruptions in serine metabolism are also seen in various chronic diseases affecting systemic organ functions, such as diabetes, liver diseases, cancer, and chronic kidney disease (CKD).^[41]^ Research in both humans and animals has shown that L-serine levels significantly decrease in the bloodstream of CKD patients and rats. Notably, the concentration of D-serine, a chiral form of L-serine, increases in CKD patients and is now recognized as a biomarker indicating the progression of the disease.^[42]^ Therefore, we propose that L-serine could serve as a protective factor against IgAN through the following mechanisms: First, studies by Rodriguez and colleagues have highlighted that serine metabolism regulates macrophage inflammatory responses both in vitro and in vivo.^[43]^ They discovered that serine metabolism is essential for producing glutathione, which is necessary for LPS-induced IL-1β mRNA expression. Furthermore, inhibiting serine synthesis offers protection in a mouse model of endotoxemia. Additional research reveals that in IgAN patients categorized as having impaired renal function and normal renal function, plasma glutathione peroxidase activity is drastically reduced to just 17% and 25% of that observed in healthy controls, respectively. These patients also exhibit increased oxidative stress and heightened inflammatory responses.^[44]^ Consequently, L-serine could play a crucial role in boosting glutathione levels, offering antioxidant and anti-inflammatory benefits that potentially reduce kidney damage in IgAN patients. Additionally, in IgAN patients, the infiltration of FoxP3 + mononuclear cells is observed in the renal interstitium.^[45]^ Therefore, L-serine may modulate T cell function and affect cytokine production, thereby mitigating kidney damage caused by abnormal immune responses. Moreover, studies indicate that L-serine could enhance vasodilation by stimulating SKCa and IKCa channels on endothelial cells. In IgAN, renal arteriosclerosis contributes to microvascular narrowing, which in turn causes glomerular ischemia and triggers the renin-angiotensin system.^[46]^ Employing renin-angiotensin system inhibitors during follow-up has been shown to improve kidney outcomes. Therefore, L-serine could potentially protect the kidneys by enhancing nitric oxide production and improving renal microcirculation, thereby reducing the damage caused by glomerular hypertension and hyperfiltration. Consequently, considering L-serine as a potential protective agent, it is valuable to investigate whether exogenous supplementation could mitigate the onset or progression of IgAN. This is particularly relevant for high-risk groups, including individuals with genetic predispositions and elevated levels of circulating Gd-IgA1.

The widely accepted multiple-hit hypothesis for IgAN suggests that elevated levels of galactose-deficient IgA1 in the circulation can trigger autoantibody activation and subsequent complex formation, which deposits in the glomerular mesangium. These deposits initiate a complement cascade, stimulate the release of cytokines that promote inflammation and fibrosis, and activate the renin-angiotensin-aldosterone system, leading to IgAN. Our use of MR techniques has linked 2 metabolites with IgAN risk; one shows a positive causal effect, while the other shows a negative one. Importantly, both metabolites are amino acids. Prior metabolomic research into IgAN has indicated disruptions in amino acid metabolism linked to inflammation, antioxidant activities, coagulation, and cell apoptosis. This research contributes to understanding potential pathogenic pathways and metabolites not previously identified by metabolomics, from a genetic standpoint. However, research focused specifically on amino acid metabolism in IgAN is still in its early stages and remains limited.

This study boasts a significant advantage, including a broad array of 1400 metabolites and employing MR to systematically explore the connections between blood metabolites and IgAN. It rigorously applies statistical techniques to minimize confounders and reduce the risk of reverse causality. However, the study faces several limitations. First, the data originate solely from Caucasian populations, which significantly restricts the generalizability of our findings to other ethnic populations, such as those of African, Asian, or Hispanic ancestry. Therefore, replication of these potential causal associations in diverse populations is crucial to ascertain their broader applicability and global health relevance. Addressing this constraint in future metabolomic MR research will be critical. While direct replication through large-scale metabolite and disease GWAS in non-European cohorts is the ideal, the current scarcity of such comprehensive data presents a challenge. Emerging strategies like trans-ethnic MR analyses and the fine-mapping of functional causal variants offer promising avenues to explore the consistency of these associations across diverse ancestries as more global datasets become available. Ultimately, elucidating conserved biological mechanisms underlying these metabolite-disease links could also bolster the broader applicability of our findings. Until then, our conclusions are predominantly relevant to European-ancestry populations. Second, the inability to perform stratified analyses, such as by age or gender, on publicly available data might introduce confounding factors. Third, the stringent threshold of 1 × 10^−8^ used for screening exposures could result in fewer SNPs per metabolite, potentially affecting the pleiotropy analysis in MR-PRESSO. Specifically, for those relying on only 3 IVs, while overall instrument strength (*F*-statistic) might be adequate for primary IVW estimates, the power and reliability of MR-Egger regression are notably diminished. With such limited SNP counts, the MR-Egger intercept, a key indicator of directional pleiotropy, becomes unstable, and its causal effect estimates (slope) often have wide confidence intervals. Consequently, for these specific metabolites, robust inference regarding horizontal pleiotropy via MR-Egger was constrained, potentially impacting the comprehensiveness of sensitivity analyses for these select findings. While other methods like the weighted median offer some utility with fewer instruments, this inherent limitation of MR-Egger warrants caution when interpreting results for metabolites with sparse genetic instrumentation. Furthermore, given the limited research on serum metabolites in IgAN, the identified metabolites should be further validated through foundational research and clinical trials. Last, the application of a stringent FDR correction led to a notable reduction in the number of metabolites deemed significantly associated with IgAN (from 13 nominal associations to ultimately 2 after all filtering steps). While this Benjamini–Hochberg procedure (*q* < 0.05) effectively minimizes Type I errors (false positives) in the context of multiple testing inherent in a metabolome-wide screen, it may concurrently increase the risk of Type II errors (false negatives), thereby potentially obscuring true causal associations of smaller magnitude. The 2 metabolites, Acisoga and L-serine, that withstood this rigorous filtering represent the most statistically robust findings from our study, providing high-confidence candidates for future mechanistic exploration and validation.

## 5. Conclusion

Overall, this research identified 2 serum metabolites associated with the risk of IgAN. Specifically, Acisoga was found to have a significant positive causal relationship with IgAN, whereas L-serine was associated with a negative causal impact on the disease. This offers evidence of how metabolic disturbances might influence the risk of developing IgAN, warranting additional confirmation through further studies.

Supplemental Digital Content is available for this article (https://links.lww.com/MD/P498).

## Acknowledgments

We thank all the participants and investigators involved in the GWAS, as well as all the authors for their contributions to this article. We also extend our gratitude to the participants and researchers involved in the FinnGen study.

## Author contributions

**Conceptualization:** Chenxin Wang.

**Data curation:** Chenxin Wang.

**Formal analysis:** Chenxin Wang.

**Investigation:** Airong Qi.

**Methodology:** Chenxin Wang, Yanran Li.

**Project administration:** Airong Qi.

**Software:** Linyu Zhong.

**Supervision:** Denggui Luo, Yuanzhao Xu.

**Validation:** Chenxin Wang, Na Sun, Denggui Luo, Yuanzhao Xu.

**Visualization:** Chenxin Wang, Linyu Zhong.

**Writing – original draft:** Chenxin Wang.

## Supplementary Material
