# Supplementary material for: Association between 1400 metabolites and IgA nephropathy: A Mendelian randomization analysis
Source: Medicine (Baltimore). 2025 Jul 25;104(30):e43353. doi: 10.1097/MD.0000000000043353 (PMC12303432; doi:10.1097/MD.0000000000043353)

Supplementary Figure 1. forest plot indicating the three metabolites as protective factors for IgAN in a MR study with statistical significance (P < 0.05).


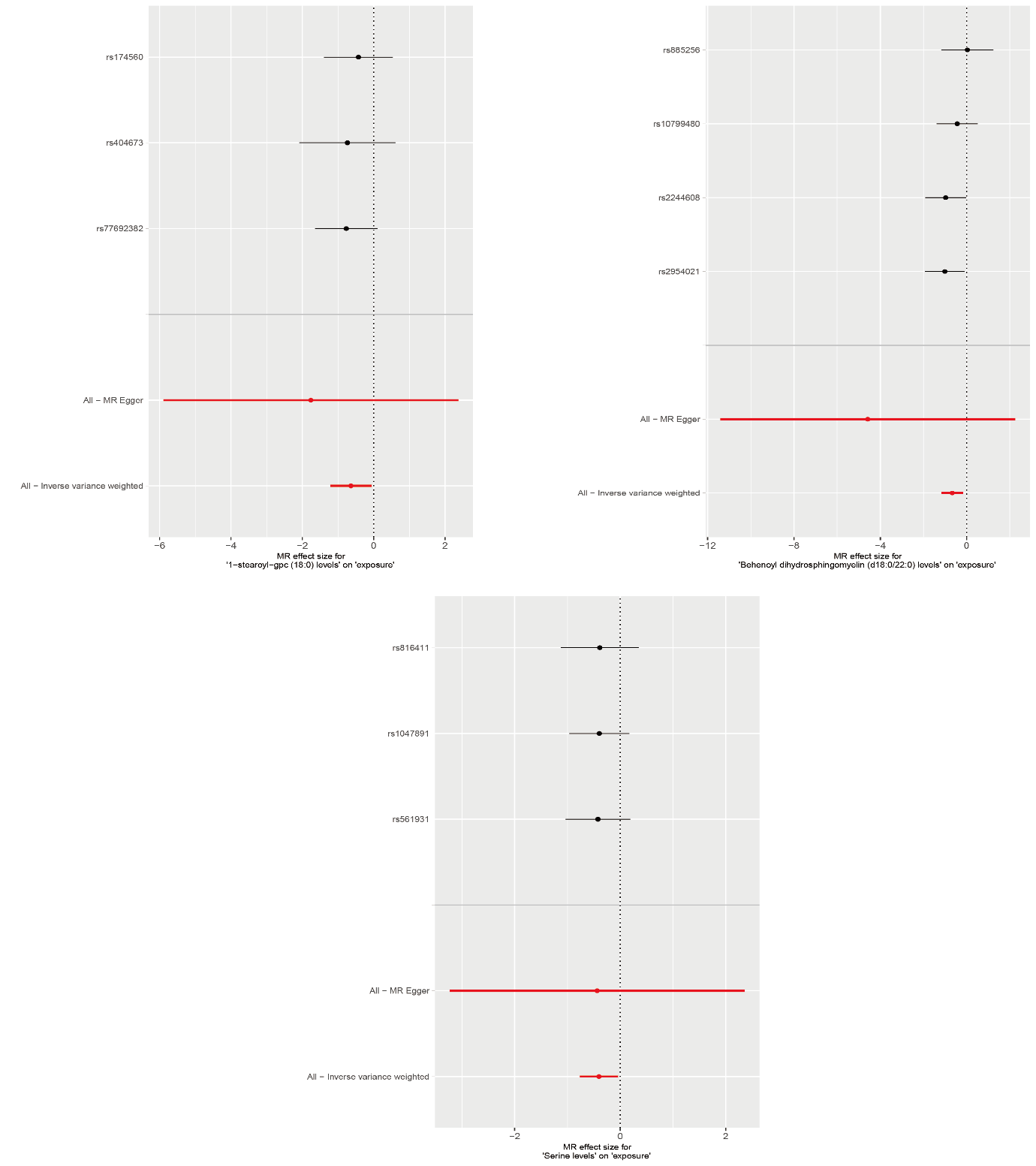


Supplementary Figure 2. forest plot indicating the six metabolites as risk factors for IgAN in a MR study with statistical significance (P < 0.05).


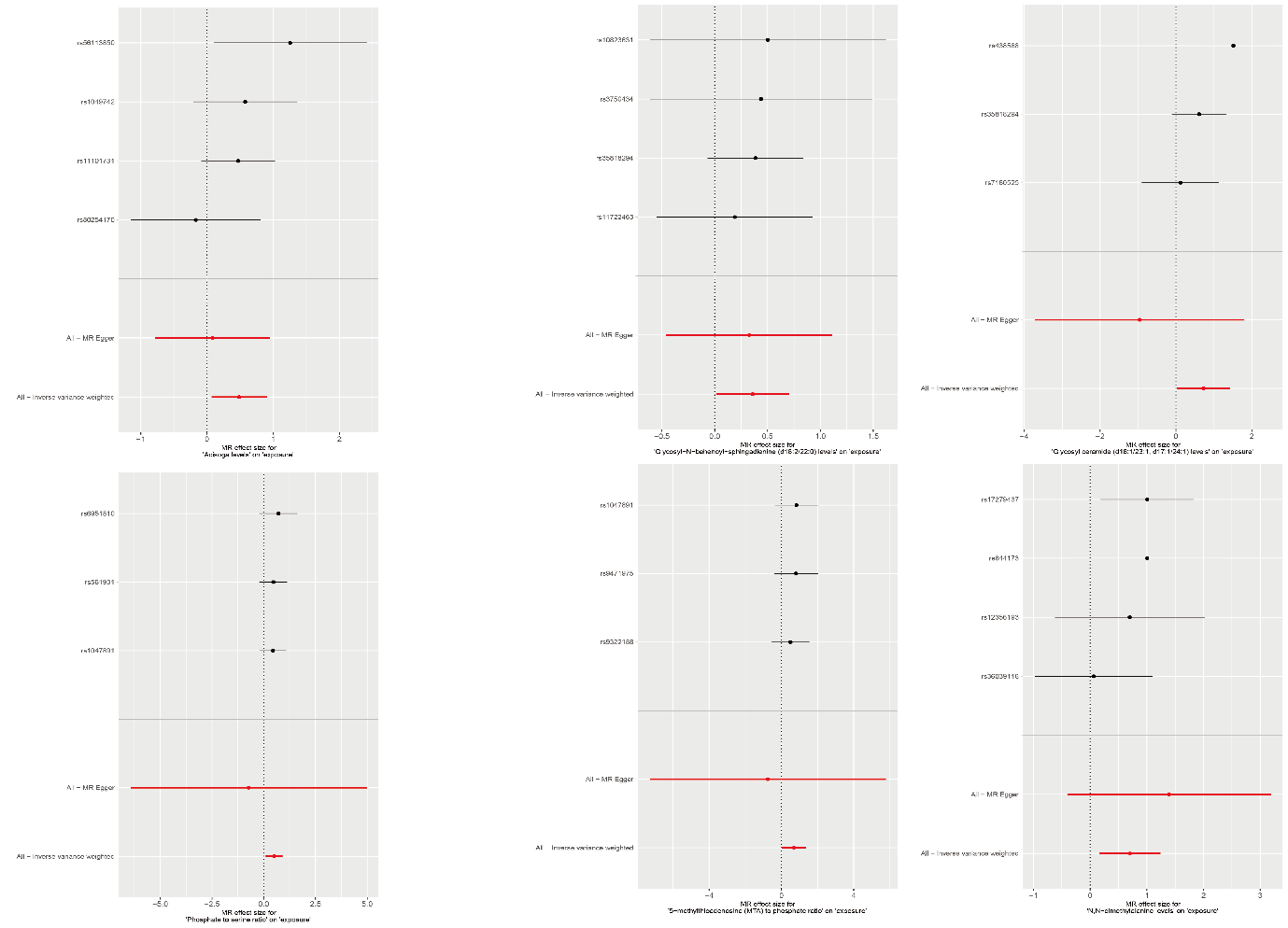


Supplementary Figure 3. leave one out plot indicating the six metabolites as risk factors for IgAN in a MR study with statistical significance (P < 0.05).


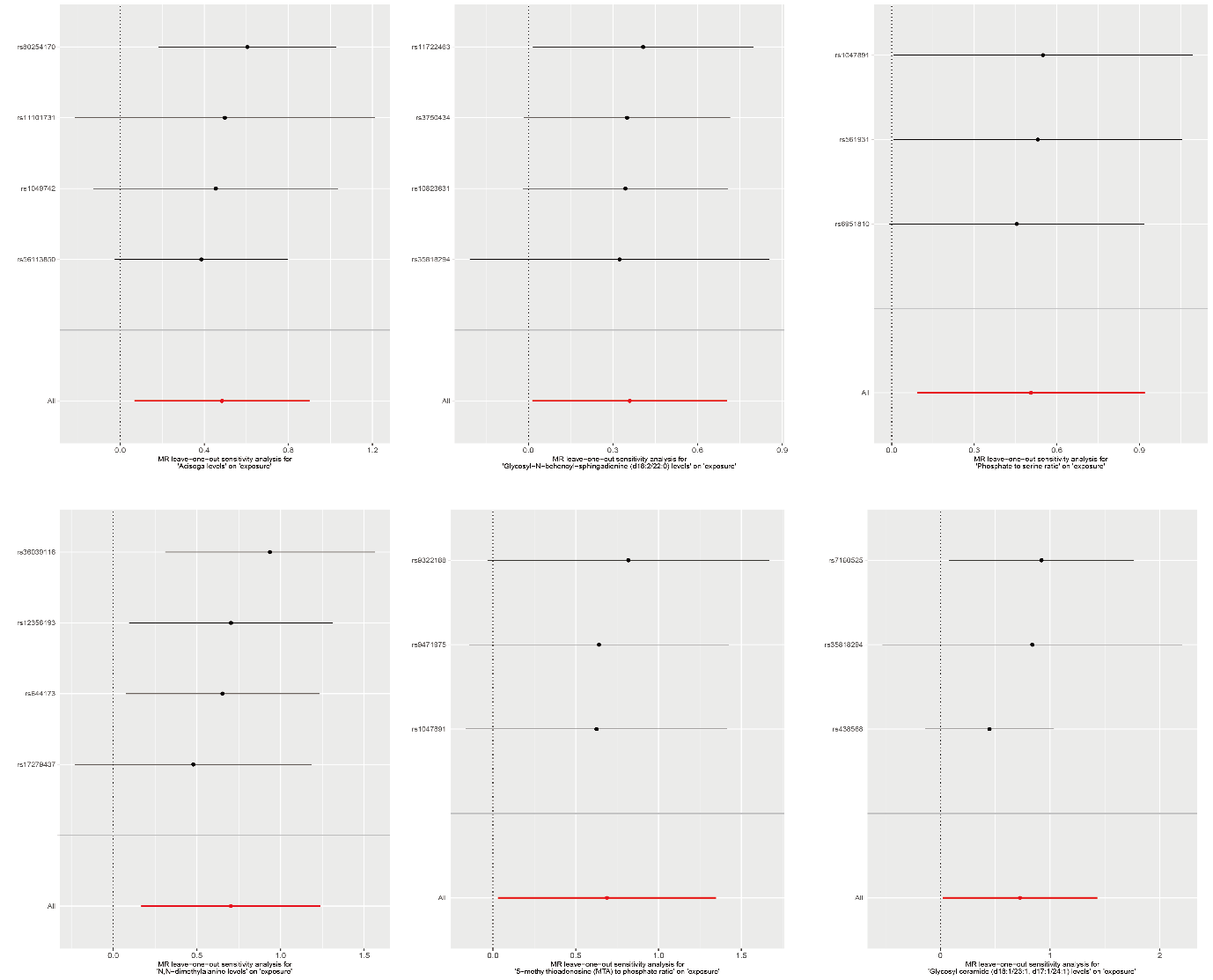


Supplementary Figure 4. leave one out plot indicating the six metabolites as protective factors for IgAN in a MR study with statistical significance (P < 0.05).


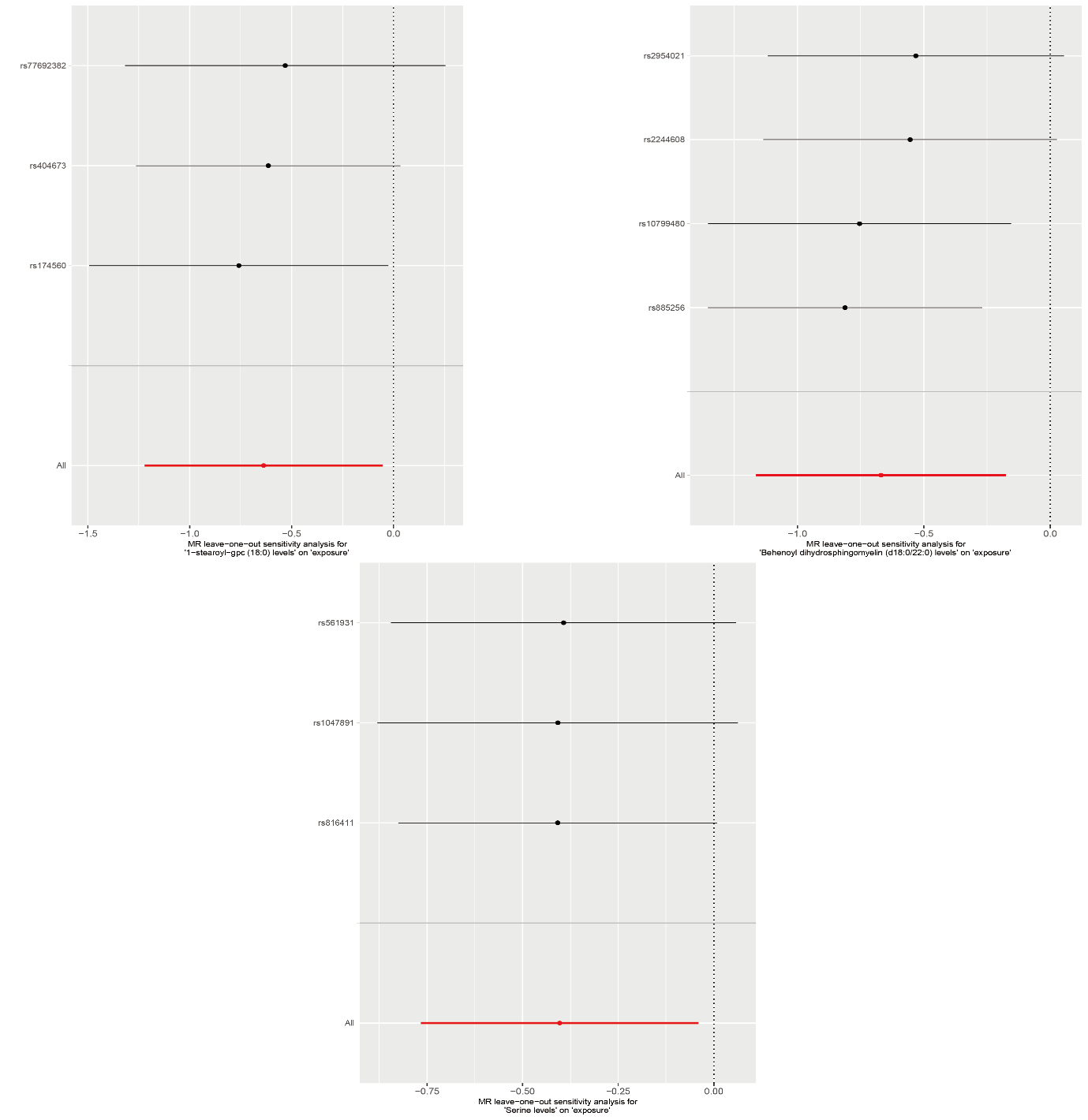


Supplementary Figure 5. Comparison of unknown metabolites using various Mendelian randomization methods


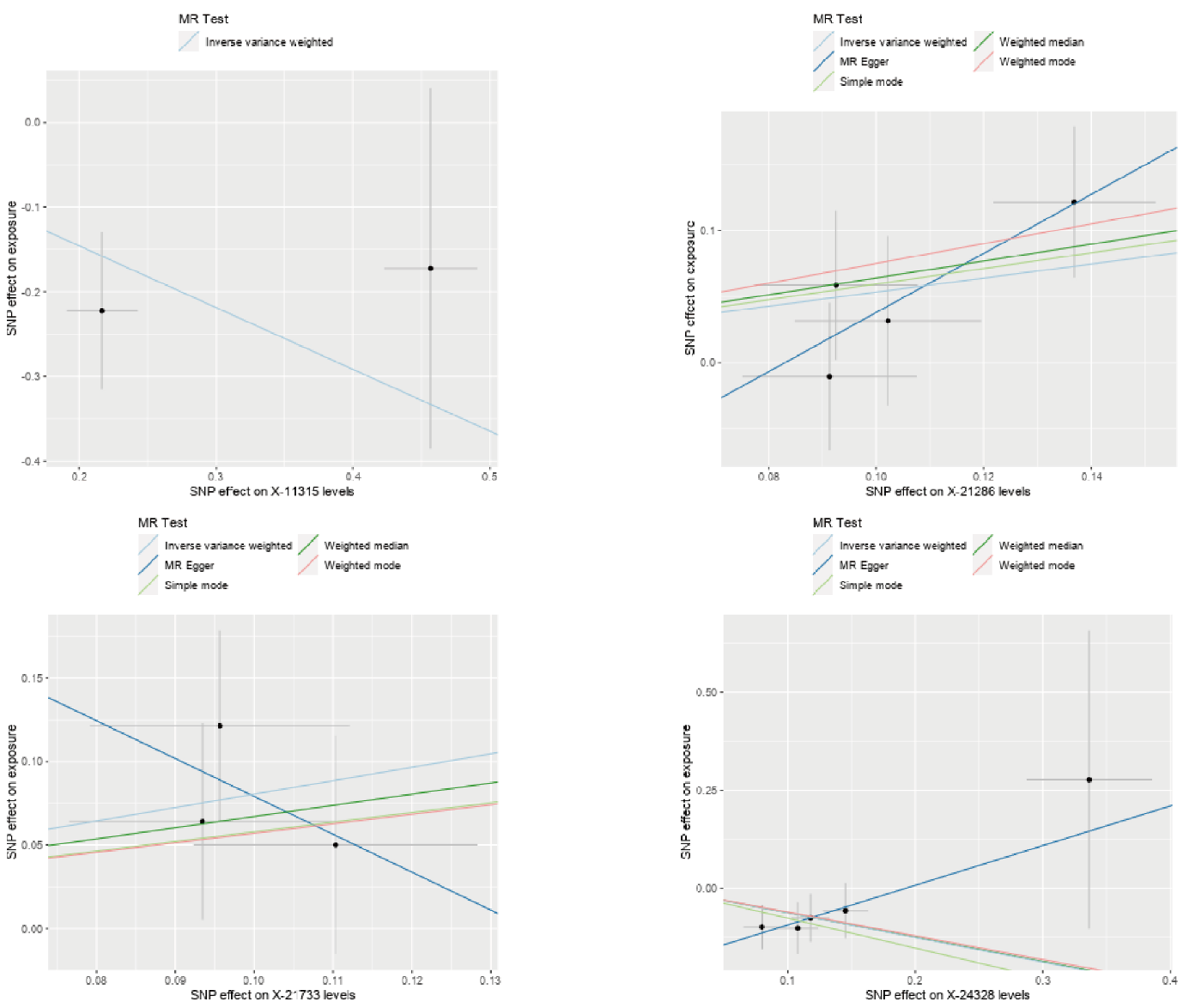

Supplement: Supplementary file 2 [file medi-104-e43353-s002.docx]
